# Chinese Graduate Health Science Students′ Perceptions of Teaching Pedagogies on Their Educational Experience

**DOI:** 10.1155/oti/5961077

**Published:** 2025-12-28

**Authors:** Jeryl D. Benson, Richard C. Simpson

**Affiliations:** ^1^ Occupational Therapy Department, Duquesne University, Pittsburgh, Pennsylvania, USA, duq.edu

**Keywords:** culturally relevant teaching, international students, learner-centered approach

## Abstract

This paper explores pedagogical approaches for teaching Chinese graduate health science students by identifying students′ perceptions of teaching and learning strategies that support learning. This study used a survey design to answer the research question: “How do Chinese English as a Foreign Language (EFL) graduate health science students perceive the impact of teaching pedagogies on their educational experience?” Descriptive methods and the Friedman test were used to analyze the data. Data from 34 completed surveys revealed that students perceive responsive instruction and guided feedback (clear, frequent, verbal, and written) as most beneficial to learning. Advance access to notes and materials also supported learning. Student‐led oral discussions and nonverbal communications (via student response systems) were deemed less beneficial. Students valued the opportunity to learn and practice clinical skills in the classroom and the clinic. These findings highlight the need for Western instructors to modify teaching strategies to meet the needs of the EFL learner while maintaining the rigor of the health science curriculum content.

## 1. Introduction

In East Asian countries, a higher education degree from an international university is related to social status and professional opportunities [1]. This has led to an increase of international students into US institutions [2], and the past decade has seen an increase in Asian students enrolling in health science graduate programs [3]. Understanding Asian cultural influence (such as high expectations and a strong work ethic) on learning is important for Western educators to meet the needs of East Asian students [1]. An awareness of relevant cultural expectations helps the instructor to better understand the challenges (such as the impact of language barriers, feelings of isolation, and mental health pressures) students face inside and outside of the classroom [4, 5]. An awareness of cultural expectations also helps the instructor to teach using pedagogical approaches that recognize cultural and language differences while maintaining standards of the health science curriculum.

## 2. Review of Literature

Asian English as a Foreign Language (EFL) students who attend university while residing in the United States face many challenges as they navigate the American higher education system. These challenges may be related to cultural adjustments, academic challenges, and nonacademic challenges. EFL students choose to live and learn in a context where their second language predominates. Attending school in another country and being taught in your nonnative language comes with some expected barriers. Some challenges to learning include difficulty with written and spoken language, increased time completing reading and written assignments, and communication from the instructor (rate of speech, pronunciation). Listening to lectures, participating in discussions, doing oral presentations, and completing learning activities in a nonnative language add a level of difficulty to comprehension and the ability to generate questions [6]. Due to the language barrier and the challenges of comprehending lectures, Asian students often rely on audio recordings of class sessions for replay after class [7]. In addition to the previous challenges mentioned, Asian students also report difficulty adjusting to the American style of teaching which is often student‐centered with an emphasis on student participation and critical thinking, as opposed to Asian educational culture which is often teacher‐focused, with the role of the student being to listen, memorize, understand, and reproduce information [8].

Asian students are often quiet in the classroom and demonstrate a lower level of participation. Choi and Nieminen suggested that the lack of speaking up in class is related to the higher level of language used in a university classroom setting and the need for the student to translate what was heard into their primary language, formulate a response, and then translate the response into English [1]. This process makes it difficult for them to actively participate in class. Restricted classroom participation due to language barriers has been linked to increased stress levels and a lack of confidence [9, 10]. Asian students report feelings of isolation and intimidation as EFL students in the classroom [11].

Culture describes values, beliefs, and social practices associated with a group of people in society [12]. Culturally based differences, between American culture and Asian culture, can lead to difficulty adapting [6]. Xiong and Zhou report that daily communication is often limited as many students report a lack of confidence in extending verbal interactions beyond a greeting and that American social conversations are structured differently and are more casual than Asian social interactions [10]. The use of slang, culturally specific jokes, and the fast pace of conversations make interactions with American students and faculty stressful, as Asian students often feel confused and uncomfortable, thereby shortening the verbal interaction [10]. The cultural differences lead to limited development of friendships and social opportunities which in turn resulted in decreased emotional support. Asian students experience stress, frustration, loneliness, anxiety, and at times depression, that is difficult for others, both in America and at home, to understand [10, 13]. In addition to the mental difficulties that stem from the cultural differences, students report stress related to finances. The high cost of studying at a US university often requires families to acquire debt to support them, resulting in feelings of guilt and added pressure to succeed [10]. International students may need to seek support provided by the university to address the cultural and mental challenges of being an EFL student. While students may expect to rely on course instructors with regard to learning challenges, few instructors have received training related to best practices of educating international students [6]. Evidence supports using teaching strategies that include increased contextual information, offering specific study approaches and increasing interaction and participation in the classroom [13, 14]. Smith et al. suggest considering how students are assessed, the structure of assignments, communication style, and lecture design and delivery, among others [13]. To further understand the needs of the international learner, it is important to ask the student what pedagogical techniques most support their learning. This study was aimed at identifying teaching strategies that have the highest student‐perceived impact on learning. To do so, we examined EFL student perceptions of various teaching approaches and the influence on their learning. The research question was “how do Chinese EFL graduate health science students perceive the impact of teaching pedagogies on their educational experience?”

## 3. Methods

### 3.1. Research Design

This study was granted an exempt status by the university Institutional Review Board (IRB). Investigators used a researcher‐designed survey to discover the perceived value of pedagogical methods on the learning of Chinese EFL students. This study used convenience sampling of students currently enrolled in a graduate health science program. Students were recruited via an email with a link to the survey. The survey was completed via Qualtrics and could be completed on either a computer or a mobile device. Study information was provided in the email sent to students and on the first page of the survey. Participants provided consent by checking a consent box and clicking “next” on the survey′s information page.

### 3.2. Research Instrument

The first author completed a focus group that included four EFL students. The focus group was an interactive discussion exploring student experiences with teaching pedagogies, including but not limited to lecture style and supports, academic assignments, and lab activities. Based on the reported experiences, the researchers designed a 58‐item survey specifically to gather data for this study (Appendix A). The survey had three parts. First, it gathered demographic information (4 items) including prior degrees earned and current program of study (physical therapy [PT], occupational therapy [OT], or speech language pathology [SLP]). The second part of the survey asked respondents to use a Likert scale to rate the following influences on learning: instructor behaviors (17 items), learning materials (14 items), and learning activities/assignments/assessments (18 items). Lastly, open‐ended, short‐answer questions (5 items) asked students to identify what contributed most and least to learning and what would improve their learning experience. Both quantitative and qualitative data were collected. The survey required approximately 20 min to complete. Qualtrics, an online survey data collection instrument, was used for this study. Participation was voluntary and anonymous. The survey was available to participants during the summer of 2022 and the summer of 2023.

### 3.3. Data Analysis

All data analyses were conducted using RStudio. The Shapiro–Wilk test was used to determine whether data were normally distributed. Data that were not normally distributed were analyzed using nonparametric statistical tests. Missing responses were assumed to be zero.

Statistical significance was defined as *p* < 0.05.

Demographic data (Q1) were summarized using descriptive methods. Nominal data were summarized using the frequency of each category. Ratio data that was not normally distributed were summarized using the minimum, maximum, and median.

Student responses were compared within each category (Q2, behaviors; Q3, materials; Q4, activities). The data were not normally distributed, so all comparisons were conducted using the Friedman test (which is similar to the repeated measures ANOVA). Post hoc comparisons were performed using the Nemenyi test, which corrects for multiple comparisons.

A secondary analysis was performed to compare students who attended classes in person (*n* = 27) and students who attended classes either partially or completely online (*n* = 7). Once again, the data was not normally distributed so differences between groups were analyzed using the Mann–Whitney *U* test (which is similar to an independent *t*‐test). We did not correct for multiple comparisons.

Text‐based survey data from the open‐ended questions were analyzed using cross‐sectional and categorical indexing beginning with a literal and interpretive review of the open‐ended responses from the survey and culminating in the development of categories [15]. Categories were developed based on repetitive patterns or regularities examined in the data as well as reflection upon similarities and differences of each data point. Triangulation occurred by the emergence of the same categories from both the open‐ended probes and the responses garnered from the survey.

## 4. Results

### 4.1. Demographic Data (Q1)

As shown in Table 1, 34 students completed the survey. Participants′ ages ranged from 22 to 35, with a median age of 25. Participants were all students enrolled in graduate health science program at Duquesne University. A total of 48 Chinese graduate students enrolled in an OT, PT, or SLP program opened the survey. Researchers did not include incomplete surveys (*n* = 14), resulting in a total of 34 completed surveys for analysis.

**Table 1:** Demographic data for survey respondents (*N* = 34).

Gender	
Male	11 (32.4%)
Female	23 (67.6%)
Attendance	
Entirely in‐person	27 (79.4%)
Hybrid	3 (8.8%)
Entirely online	4 (11.8%)
Program of study	
Physical therapy	18 (52.9%)
Occupational therapy	9 (26.5%)
Speech language pathology	7 (20.6%)
Age	
Min	22
Max	35
Median	25

Students were OT (26.5%), SLP (20.6%), and PT (52.9%). Most respondents (79.4%) attended classes in person, but some attended either entirely (11.8%) or partly (hybrid, 8.8%) online. The age of students was 20–35 years with most identifying as female (67.6%).

### 4.2. Instructor Behaviors (Q2) (See Table 2)

**Table 2:** Median student response for Q2 (instructor behaviors): These are things the instructor did while actively teaching to deliver course content. Indicate how much each of the following instructor behaviors contributed to your learning.

**Item**	**Median student response**
Provide written/verbal feedback	80
Provides step by step instructions	80
Uses video case studies to apply content, explanation of concepts during video case	80
Uses paper case studies to apply content, instructor explanation of concepts using the case study	80
Instructor to student class communications (class announcements/group emails)	80
Instructor to student individual communications (personal emails)	75
Instructor use of body language/use of gestures	75
List/spell/define/repeat new words	75
Ask students questions	70
Asks students to restate ideas	70
Instructor office hours/individual student meetings	65
Instructor permitted students to speak in their native language during classroom learning activities	60
Volume of instructors′ speech (how loud they talk)	60
Extended wait time for processing	60
Rate of instructors′ speech (how fast they talk)	55
Repeating and/or paraphrasing	55
Ask student to respond nonverbally (Kahoot, Nearpod, Student Response System)	50

The Friedman test identified a statistically significant difference in student responses for instructor behaviors (*p* = 0.00007). The post hoc Nemenyi test identified the following statistically significant pairwise differences:

• Instructor to student class communications (class announcements/group emails) [median = 80] versus
  o. Rate of instructor′s speech (how fast they talk) [median = 55] (*p* < 0.05)
  o. Ask student to respond nonverbally (Kahoot, Nearpod, Student Response System) [median = 50] (*p* < 0.01)

Several pairwise differences approached, but did not reach, statistical significance:

• Instructor to student class communications (class announcements/group emails) [median = 80] versus
  o. Instructor office hours/individual student meetings [median = 65] (*p* = 0.09656)
  o. Extended wait time for processing [median = 60] (*p* = 0.07544)
• Ask student to respond nonverbally (Kahoot, Nearpod, Student Response System) [median = 50] versus
  o. Provide written/verbal feedback [median = 80] (*p* = 0.05403)
  o. Provides step by step instructions [median = 80] (*p* = 0.09993)
  o. Uses video case studies to apply content, explanation of concepts during video case [median = 80] (*p* = 0.0782)

### 4.3. Course Materials (Q3) (See Table 3)

**Table 3:** Median student response for Q3 (materials used for instruction): These are the resources instructors used to support learning. Indicate how much each of the following materials contributed to your learning.

**Item**	**Median student response**
Presentation slides include diagrams, images, and graphics	100
Presentation slides/class notes provided in advance	95
Lectures are recorded for student access outside of class	95
Online learning platform such as Blackboard or Canvas	90
Presentation slides/class notes provided in class	90
Presentation slides visually emphasize key concepts by highlighting key words/phrases	90
Presentation slides include bulleted lists	90
Videos/movies	85
Research databases	80
Textbooks	80
Research articles	80
Online resources (such as websites)	80
White board	75
University library	70

The Friedman test identified a statistically significant difference in student responses for instructor behaviors (*p* < 0.001). The post hoc Nemenyi test identified the following statistically significant pairwise differences:

• University library [median = 70] versus
  o. Online learning platform such as Blackboard or Canvas [median = 90] (*p* < 0.01)
  o. Presentation slides/class notes provided in advance [median = 95] (*p* < 0.001)
  o. Presentation slides/class notes provided in class [median = 90] (*p* < 0.01)
  o. Presentation slides include diagrams, images, and graphics [median = 100] (*p* < 0.001)
  o. Presentation slides visually emphasize key concepts by highlighting key words/phrases [median = 90] (*p* < 0.001)
  o. Lectures are recorded for student access outside of class [median = 95] (*p* < 0.01)
• Research databases [median = 80] versus
  o. Online learning platform such as Blackboard or Canvas [median = 90] (*p* < 0.05)
  o. Presentation slides/class notes provided in advance [median = 95] (*p* < 0.01)
  o. Presentation slides include diagrams, images, and graphics [median = 100] (*p* < 0.01)
  o. Presentation slides visually emphasize key concepts by highlighting key words/phrases [median = 90] (*p* < 0.01)
  o. Lectures are recorded for student access outside of class [median = 95] (*p* < 0.05)
• White board [median = 75] versus presentation slides include diagrams, images, and graphics [median = 100] (*p* < 0.05)

Several pairwise differences approached, but did not reach, statistical significance:

• University library [median = 70] versus
  o. Presentation slides include bulleted lists [median = 90] (*p* = 0.05086)
  o. Online resources (such as websites) [median = 80] (*p* = 0.0664)
  o. Videos/movies [median = 85] (*p* = 0.06356)
• White board [median = 75] versus
  o. Presentation slides/class notes provided in advance [median = 95] (*p* = 0.06934)
  o. Presentation slides visually emphasize key concepts by highlighting key words/phrases [median = 90] (*p* = 0.08933)
• Research databases [median = 80] versus presentation slides/class notes provided in class [median = 90] (*p* = 0.05566)
• Textbooks [median = 80] versus presentation slides include diagrams, images, and graphics [median = 100] (*p* = 0.0664)

### 4.4. Activities (Q4) (See Table 4)

**Table 4:** Median student response for Q4 (learning activities/assignments/assessment): These were the assignments and activities the instructor assigned to you to facilitate and assess your learning. Indicate how much each of the following learning activities or assessments contributed to your learning.

**Item**	**Median student response**
Study guides	90
Instructor demonstration of therapeutic equipment use such as assessment tools, weights, and therapy balls	90
Student opportunity to practice with and use therapeutic equipment	90
Clinical observations of therapy practice in the community	90
Student analysis of paper case studies	80
Practice exams/sample exam questions	80
Oral presentations	80
Small group work/working with a partner	80
Comprehensive exam/practical exams/student demonstration of skills	80
Assignments that required a draft and offered feedback	80
Written exams	80
Quizzes	80
Term paper/research paper	80
Treatment plans/intervention plans	80
Student hands‐on treatment experience/practice with a client/patient supervised by the instructor	80
Graphic organizers/worksheets to structure in class activities	75
Student analysis of video case studies	70
Use of technology that allows for nonverbal/anonymous student responses (such as Kahoot, Nearpod, and Student Response System via phone or device)	65

The Friedman test identified a statistically significant difference in student responses for instructor behaviors (*p* < 0.001). The post hoc Nemenyi test identified the following statistically significant pairwise differences:

• Use of technology that allows for nonverbal/anonymous student responses (such as Kahoot, Nearpod, and Student Response System via phone or device) [median = 65] versus
  o. Treatment plans/intervention plans [median = 80] (*p* < 0.05)
  o. Instructor demonstration of therapeutic equipment use such as assessment tools, weights, and therapy balls [median = 90] (*p* < 0.01)
  o. Student opportunity to practice with and use therapeutic equipment [median = 90] (*p* < 0.01)
  o. Clinical observations of therapy practice in the community [median = 90] (*p* < 0.05)

Several pairwise differences approached, but did not reach, statistical significance:

• Term paper/research paper [median = 80] versus
  o. Instructor demonstration of therapeutic equipment use such as assessment tools, weights, and therapy balls [median = 90] (*p* = 0.07695)
  o. Student opportunity to practice with and use therapeutic equipment [median = 90] (*p* = 0.09107)
  o. Oral presentations [median = 80] versus instructor demonstration of therapeutic equipment use such as assessment tools, weights, and therapy balls [median = 90] (*p* = 0.09414)

### 4.5. Secondary Analysis—Students Who Attended Classes in Person Versus Hybrid/Online Students

For this analysis, students were grouped based on whether they attended classes entirely in person (*n* = 26) or not (*n* = 7). When comparing between groups, a strong pattern emerged where students who attended class partially or entirely online felt that many instructor behaviors and instructional materials were less effective than students who attended in person. Specifically, within instructor behaviors (Q2), there were statistically significant (*p* < 0.05) differences between groups for the following:

• Instructor use of body language/use of gestures [median for in‐person = 80, median for hybrid/online = 50]
• Provide written/verbal feedback [median for in‐person = 80, median for hybrid/online = 50]
• Provides step by step instructions [median for in‐person = 80, median for hybrid/online = 50]
• Asks students to restate ideas [median for in‐person = 70, median for hybrid/online = 50]
• Uses paper case studies to apply content, instructor explanation of concepts using the case study [median for in‐person = 80, median for hybrid/online = 60]
• Instructor to student class communications (class announcements/group emails) [median for in‐person = 80, median for hybrid/online = 50]

Within the instructional materials (Q3) category, there were statistically significant differences for the following:

• Presentation slides/class notes provided in advance [median for in‐person = 100, median for hybrid/online = 80]
• Presentation slides include diagrams, images, and graphics [median for in‐person = 85, median for hybrid/online = 70]
• Presentation slides visually emphasize key concepts by highlighting key words/phrases [median for in‐person = 85, median for hybrid/online = 70]

There were no instructional behaviors, instructional materials, or classroom activities which were considered more effective by the hybrid and fully online students.

The open‐ended prompts resulted in a wide variety of responses. However, there were a few categories that emerged for each prompt. The prompt of “what contributed most to your learning” identified the hands‐on lab experiences guided by the instructor as the most beneficial to learning. Respondents also identified their own English language proficiency as a primary contributor to learning. Respondents identified the use of videos, although very helpful to contextualize clinical interactions, as a challenge to their learning. One student stated that videos without subtitles are very hard to follow. Another student shared that engaging in English (responding orally or writing on the board) was difficult as the focus for the learner was their English ability and not their understanding of the content. The final open‐ended question asked the respondents to identify what would improve their learning experience. The overwhelming response was the opportunity for clinical observations or hands‐on clinical experiences with clients as this provides an opportunity to combine knowledge and practice. In addition, the opportunity for more classroom learning activities and supervised labs to allow the practice of newly learned clinical skills. This is consistent with the survey responses as clinical experiences, classroom learning activities, and hands‐on lab experiences provide an opportunity for responsive instruction and guided feedback.

## 5. Discussion

The results indicate that many instructional practices that are known to be effective for in‐person instruction might need to be altered for use in online instruction. These results should be interpreted with caution, because this was a secondary analysis and the number of students in the hybrid/online group was small (*n* = 7) relative to the in‐person group (*n* = 27).

Overall, outcomes from this study are consistent with current evidence. Instructor behaviors that support student learning centered on communication, specifically responsive instruction and guided feedback. Clear instructor communication was the main behavior identified by students. Students benefit from written and verbal feedback, clearly outlined instructions related to learning activities and assignments, and frequent email communications and reminders to ensure student progress on assignments meets the instructor requirements. This is consistent with the findings reported by Loh and Teo that Asian students expect instructors to provide structure and guidance for completion of assignments and learning materials [8]. Instructor behaviors during lecture that did not support learning were language‐based. Western‐based instructional design often focuses on increasing student engagement using tools requiring a nonverbal response from students (such as student response systems) or eliciting classroom discussion [8]. EFL students did not identify these techniques as valuable. Students may have difficulty understanding the prompt in English and then being required to respond quickly in English. This type of learning activity does not allow the EFL student time to translate and formulate a meaningful response [6]. Students may prefer to have time to process information and email questions or ask questions after class when the exchange is just between the learner and the instructor [8].

The students identified the instructor′s demonstration of clinical techniques and skills followed by the opportunity to practice those skills as one of the most beneficial learning activities. This was followed by clinical observation of clients which helped to put the pieces together. Students also identified the use of case studies to support learning. However, text data indicated that videos would be more supportive of learning if subtitles were included.

Learning materials that supported student success include having a Learning Management System that houses classroom resources such as notes, readings, and recordings of lectures. Students identified that having notes that are visually organized for clarity (instructor should consider language use, writing out acronyms, and use of graphs and tables) in advance helped with class preparation. Consistent with current evidence, access to the recorded lecture after class to review was identified as a main support to learning [7].

There is currently a lack of literature related to Asian EFL students studying health sciences in the United States. A clearer understanding of how Asian EFL learners acquire clinical knowledge is important for developing effective teaching strategies. This understanding would also support the design of a culturally supportive learning environment [16]. This research identified multiple learner preferences and supports for learning for the Asian EFL student. When designing and delivering a course, culturally relevant learning tools (materials, assignments, etc.) should be considered. The role and behaviors of the instructor were identified as a key factor in creating a culturally supportive learning environment. This study highlights the importance of immersive learning in a clinical context to create connections and provide meaning. In addition, it proposes the use of guided and corrective feedback to support understanding as learning is occurring. Lastly, the use of written feedback allows the learner time for processing language differences and the opportunity for reflection.

## 6. Limitations and Future Research

Outcomes from this study should be generalized with caution as there were multiple limitations. First, the survey tool was created for this study and was written in English; therefore, it does not have established reliability or validity. Second, the sample size was small and represents a group of Chinese students enrolled at one American university pursuing a health science degree. Third, the students were enrolled in one of three programs, and there may be differences in instructor and/or program style. Another potential limitation was that the survey was not culturally vetted. Finally, limitations related to the survey design include the length of the survey, time commitment to complete the survey, and potential technological issues.

## 7. Conclusion

Health science education is experiencing an increase in international students seeking graduate health science degrees from an American university. Universities and faculty must be prepared to teach rigorous content in culturally relevant and meaningful ways to ensure learning. Graduate health science programs can use these outcomes to identify learning differences based on culture and tailor the educational experience to meet the needs of the learner. When teaching international students, cultural and pedagogical differences are inevitable. More studies are needed to understand how to better prepare faculty.

## Conflicts of Interest

The authors declare no conflicts of interest.

## Funding

No funding was received for this manuscript.

## Data Availability

The data that support the findings of this study are available from the corresponding author upon reasonable request.

## Appendix A

**Table A1:** Survey rated on a 10‐point Likert scale from “none at all” to “a great deal.” If a strategy was not used, students were asked to indicate this statement is not applicable.

Instructor behaviors: These are things *the instructor did* while actively teaching to deliver course content. Indicate how much each of the following instructor behaviors *contributed your learning* :	Rate of instructors′ speech (how fast they talk)
	Volume of instructors′ speech (how loud they talk)
	Instructor use of body language/use of gestures
	Repeating and/or paraphrasing
	Extended wait time for processing
	List/spell/define/repeat new words
	Ask students questions
	Provide written/verbal feedback
	Provides step by step instructions
	Asks students to restate ideas
	Ask student to respond nonverbally (Kahoot, Nearpod, and Student Response System)
	Uses video case studies to apply content, explanation of concepts during video case
	Uses paper case studies to apply content, instructor explanation of concepts using the case study
	Instructor to student class communications (class announcements/group emails)
	Instructor to student individual communications (personal emails)
	Instructor office hours/individual student meetings
	Instructor permitted students to speak in their native language during classroom learning activities
Materials used for instruction: These are the resources instructors used to support learning. Indicate how much each of the following materials *contributed to your learning*	Online learning platform such as Blackboard or Canvas
	Presentation slides/class notes provided in advance
	Presentation slides/class notes provided in class
	Presentation slides include diagrams, images, and graphics
	Presentation slides visually emphasize key concepts by highlighting key words/phrases
	Presentation slides include bulleted lists
	Lectures are recorded for student access outside of class
	White board
	Textbooks
	Research articles
	Online resources (such as websites)
	Videos/movies
	University library
	Research databases
Learning activities/assignments/assessment: These were the assignments and activities the instructor assigned to you to facilitate and assess your learning. Indicate how much each of the following learning activities or assessments *contributed your learning*	Student analysis of paper case studies
	Student analysis of video case studies
	Oral presentations
	Small group work/working with a partner
	Comprehensive exam/practical exams/student demonstration of skills
	Assignments that required a draft and offered feedback
	Written exams
	Quizzes
	Term paper/research paper
	Treatment plans/intervention plans
	Graphic organizers/worksheets to structure in class activities
	Use of technology that allows for nonverbal/anonymous student responses (such as Kahoot, Nearpod, and Student Response System via phone or device)
	Study guides
	Practice exams/sample exam questions
	Instructor demonstration of therapeutic equipment use such as assessment tools, weights, and therapy balls
	Student opportunity to practice with and use therapeutic equipment
	Clinical observations of therapy practice in the community
	Student hands‐on treatment experience/practice with a client/patient supervised by the instructor

*Note:* Open‐ended questions: What contributed most to your learning? Why? What contributed least to your learning? Why? What, if anything, would improve your learning experience? Please share your thoughts about your time as a student in this program.
